# High‐Pressure Synthesis of Nitrogen‐Rich Y(N_5_)_3_·N_2_ Pentazolate with Perovskite Topology

**DOI:** 10.1002/anie.202506334

**Published:** 2025-06-16

**Authors:** Andrey Aslandukov, Yuqing Yin, Maxim Bykov, Alena Aslandukova, Fariia I. Akbar, Eleanor Lawrence Bright, Igor A. Abrikosov, Natalia Dubrovinskaia, Leonid Dubrovinsky

**Affiliations:** ^1^ Bayerisches Geoinstitut University of Bayreuth Universitätstrasse 30 Bayreuth 95440 Germany; ^2^ Material Physics and Technology at Extreme Conditions Laboratory of Crystallography University of Bayreuth Universitätstrasse 30 Bayreuth 95440 Germany; ^3^ Goethe University Frankfurt Institute of Inorganic and Analytical Chemistry Max‐von‐Laue‐Straße 7 Frankfurt am Main 60438 Germany; ^4^ Department of Physics Chemistry and Biology (IFM) Linköping University Linköping SE‐581 83 Sweden; ^5^ European Synchrotron Radiation Facility CS 40220 Grenoble Cedex 9 38043 France

**Keywords:** Diamond anvil cell, High‐pressure, Pentazolate, Single‐crystal X‐ray diffraction

## Abstract

The discovery and stabilization of the cyclo‐N_5_⁻ anion have introduced a class of pentazolate compounds with significant potential as high‐energy‐density materials (HEDMs). This potential could be further enhanced by increasing the pentazolate‐to‐metal ratio. Here, we report the high‐pressure, high‐temperature synthesis and characterization of a novel yttrium pentazolate, Y(N_5_)_3_·N_2_, which exhibits an exceptionally high nitrogen‐to‐metal ratio of 17:1. Y(N_5_)_3_·N_2_ was synthesized from yttrium and nitrogen by laser heating to 3000 K at 125 GPa in a diamond anvil cell. Its crystal structure, a 3D nitrogen‐inclusion metal‐pentazolate framework, was solved and refined in situ using synchrotron single‐crystal X‐ray diffraction. This structure demonstrates a perovskite topology, as pentazolate ring centers form octahedra connected via vertices, neutral nitrogen dimers are located at the centers of the octahedra, and yttrium atoms occupy distorted cuboctahedra within the octahedral 3D framework. Density functional theory (DFT) calculations corroborate the experimental findings and provide further insights into the stability and properties of the synthesized compound. Y(N_5_)_3_·N_2_ is the first example of stabilizing three pentazolate anions per metal cation, surpassing the previously achieved 1:1 ratio. Additionally, we propose a centroid‐based structure typification for solvent‐free inorganic pentazolates, serving as an important step for further structural classification of pentazolates and other polynitrides.

## Graphical abstract

Yttrium pentazolate, Y(N_5_)_3_·N_2_, with an exceptionally high nitrogen‐to‐metal ratio of 17:1 and perovskite topology, was synthesized from yttrium and nitrogen at 125 GPa and 3000 K in a laser‐heated diamond anvil cell.

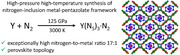

Pentazolates, featuring the cyclo‐N_5_⁻ anion, represent a fascinating class of nitrogen‐rich compounds with remarkable potential as high‐energy‐density materials (HEDMs). Although this five‐membered all‐nitrogen ring is composed of high‐energy nitrogen─nitrogen bonds, its aromaticity contributes to the stabilization of pentazolate salts. Following the first stabilization of the pentazolate anion in (N_5_)_6_(H_3_O)_3_(NH_4_)_4_Cl in 2017,^[^ ^1^ ^]^ a series of metal pentazolate hydrates, including [Na(H_2_O)(N_5_)_2_]·2H_2_O, [Mg(H_2_O)_6_(N_5_)_2_]·4H_2_O, [M(H_2_O)_4_(N_5_)_2_]·4H_2_O (M = Mn, Fe, Co, Zn), Ba(N_5_)(NO_3_)(H_2_O)_3_, and NaBa_3_(N_5_)_6_(NO_3_)(H_2_O)_3_, were synthesized.^[^ ^2^, ^3^, ^4^, ^5^ ^]^ All these salts contain the isolated cyclo‐N^5−^ together with large amounts of coordinated water molecules, which play an important role in stabilizing the *cyclo*‐N_5_⁻ anion through extensive hydrogen bonds. However, the presence of water in these salts limits their utility for HEDM applications.

There are two approaches to the synthesis of solvent‐free pentazolate salts. The first one is based on the exchange reactions with other pentazolate salts. Solvent‐free pentazolate frameworks AgN_5_ and Cu(N_3_)(N_5_) were obtained by exchange reaction of AgNO_3_ or Cu(NO_3_)_2_ with [Na(H_2_O)(N_5_)]·2H_2_O (or its mixture with NaN_3_),^[^ ^5^ ^]^ while LiN_5_,^[^ ^6^ ^]^ KN_5_,^[^ ^7^ ^]^ and NH_4_N_5_^[^ ^7^ ^]^ were synthesized by exchange reaction of corresponding XCl (X = Li, K, NH_4_) chlorides with AgN_5_. Another synthetic route to solvent‐free pentazolates is high‐pressure synthesis. A number of compounds containing pentazolate anions, such as LiN_5_, NaN_5_, NaN_5_·N_2_, K_9_N_56_, and CsN_5_, were synthesized by the reaction of nitrogen and corresponding azide in laser‐heated diamond anvil cells under pressures of 45–60 GPa and temperatures above 1200 K.^[^ ^8^, ^9^, ^10^, ^11^ ^]^ Some of these high‐pressure phases, namely LiN_5_ and CsN_5_, are reported to be recoverable to ambient conditions.^[^ ^8^, ^11^ ^]^

An important aspect in the development of pentazolates for HEDM applications is the pentazolate‐to‐metal ratio. Nowadays, among known solvent‐free metal‐pentazolate compounds the maximum pentazolate‐to‐metal ratio is 1:1. However higher metal‐to‐pentazolate ratios could significantly enhance the energy density. Although many compounds with higher metal‐to‐pentazolate ratios have been predicted using structure search algorithms,^[^ ^12^ ^]^ none have been synthesized to date.

Yttrium, as a +3 cation, can hypothetically form pentazolate salts with a pentazolate‐to‐metal ratio up to 3:1. Theoretical studies predict numerous high‐pressure, nitrogen‐rich yttrium compounds that are reported to be promising HEDMs.^[^ ^12^, ^13^, ^14^, ^15^ ^]^ Among these, YN_15_ and YN_10_ notably feature pentazolate anions.^[^ ^12^, ^13^ ^]^ In experimental studies, cubic yttrium nitride YN with a rock salt structure is well‐known under ambient conditions and is predicted to remain stable up to at least 135 GPa.^[^ ^16^ ^]^ Under moderate pressure (∼50 GPa), yttrium and nitrogen form Y_5_N_14_, which contains three types of [N_2_]^x−^ dimers.^[^ ^17^ ^]^ At 100 GPa, yttrium polynitrides YN_6_ and Y_2_N_11_ are synthesized, exhibiting unique arrangements of nitrogen atoms, including anionic N_18_ macrocycles and a polynitrogen double helix.^[^ ^18^ ^]^ To date, none of the experimentally found high‐pressure yttrium nitrides were predicted, and conversely, none of the theoretically predicted structures with promising energetic properties have been experimentally observed.

Here, we report the high‐pressure, high‐temperature synthesis and characterization of Y(N_5_)_3_·N_2_ at 125 GPa. The crystal structure of Y(N_5_)_3_·N_2_—a binary compound with an exceptionally high nitrogen‐to‐metal ratio of 17:1—features pentazolate rings and neutral nitrogen dimers. This compound represents the first example of stabilizing three pentazolate anions per metal cation.

In this study a diamond anvil cell loaded with an yttrium piece embedded in molecular nitrogen was used (see Methods section in Supporting Information for details). The sample was compressed to 125(2) GPa and laser‐heated at 3000(300) K (Figure 1a,b). Then the multigrain sample, consisting of many tiny crystallites of reaction product(s) obtained after laser heating, was studied by single‐crystal X‐ray diffraction at the ID11 beamline at ESRF with an HWHM beamsize of 0.75 × 0.75 µm^2^. First, a detailed X‐ray diffraction mapping was made within the heated spot to pinpoint the location of crystallites most appropriate for single‐crystal X‐ray diffraction measurements (Figure 1c). Then single‐crystal X‐ray diffraction data were collected at the selected positions to identify the phase, its crystal structure, and its chemical composition.

**Figure 1:**
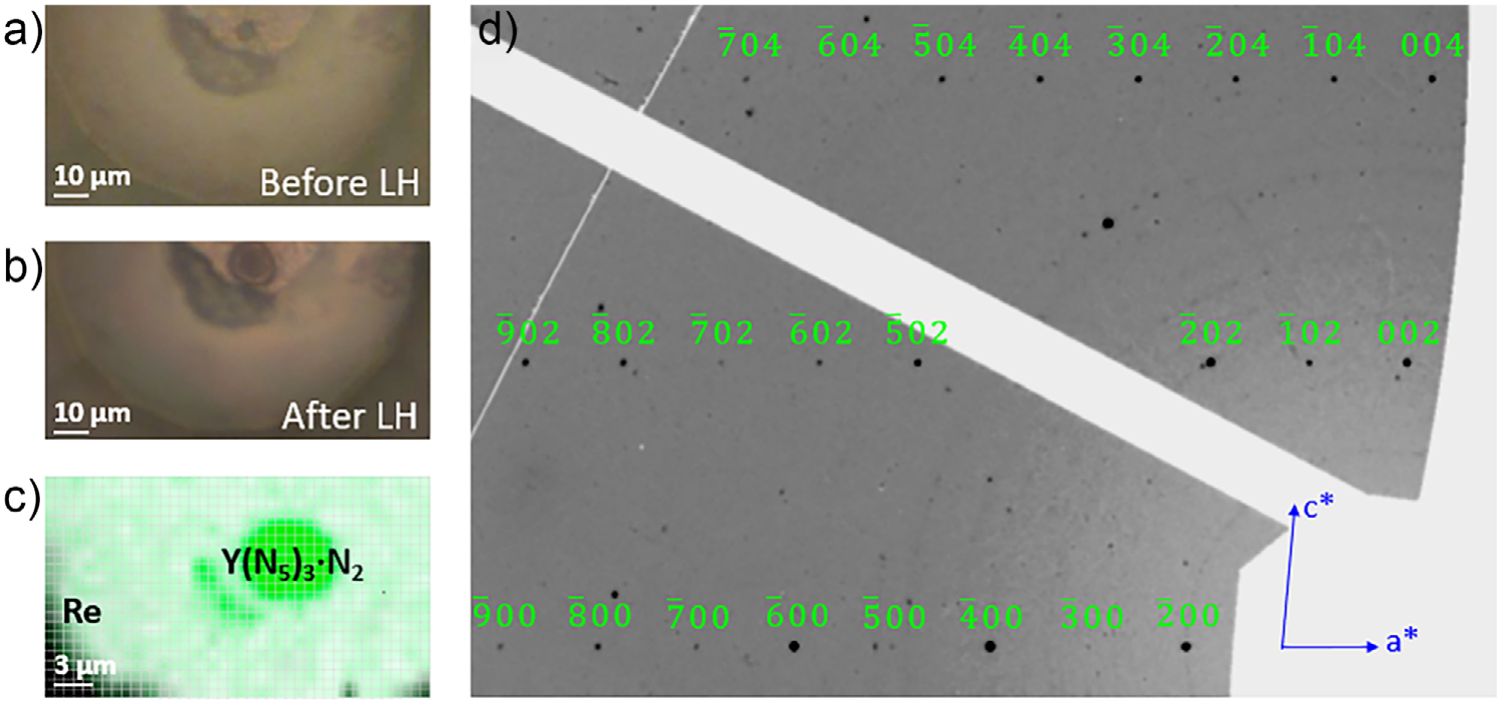
Sample characterization. a) Micro‐photo of the half of the sample chamber containing a piece of yttrium embedded in nitrogen at 125 GPa before laser‐heating and b) after laser‐heating. c) 2D X‐ray diffraction map (collected with 0.75 µm steps at the ID11 beamline of the ESRF) showing the homogeneous distribution of the Y(N_5_)_3_·N_2_ phase, as determined by single‐crystal XRD, within the heated sample at 125 GPa. d) Part of the reconstructed (*h 0 l*) reciprocal lattice plane of Y(N_5_)_3_·N_2_ at 125 GPa from the experimental single‐crystal XRD dataset obtained using CrysAlis^Pro^ software. The green‐labeled reflections correspond to the crystallite of Y(N_5_)_3_·N_2_, whose structure was determined. The *h* 0 *l* reflections with *l* = 2n + 1 are systematically absent in the *P*2_1_/*c* space group.

Analysis of synchrotron single‐crystal X‐ray diffraction data (Figure 1d) revealed the formation of a single reaction product, Y(N_5_)_3_·N_2_. Its structure has a monoclinic space group *P*2_1_/*c* (#14) with the lattice parameters *a* = 9.6169(12) Å, *b* = 6.932(5) Å, *c* = 6.6089(11) Å, and *β* = 95.134(14)° at 125(2) GPa (see Table S1 and the CIF for the full crystallographic data^[^ ^19^ ^]^). The refinement against single‐crystal X‐ray diffraction data resulted in very good agreement factors (Table S1). It was homogeneously formed within the entire heated sample (Figure 1c), with no other reaction products detected (Figure S1).

The crystal structure of Y(N_5_)_3_·N_2_ is shown in Figure 2. Y occupies one atomic position, while N occupies seventeen distinct atomic positions (see Table S4 and the CIF for the full crystallographic data^[^ ^19^ ^]^). Nitrogen atoms form three crystallographically distinct pentazolate rings and two distinct dimers. The crystal structure of Y(N_5_)_3_·N_2_, as projected along all three crystallographic directions, is shown in Figure 2a–c. It can be described as a four‐layer stacking along the *a* direction. The layers are labeled in Figure 2b–g. In crystallographically distinct layers 1 and 3 (Figure 2d,f) the pentazolate rings are oriented perpendicular to the *bc* plane, while the nitrogen dimers intersect the *bc* plane at angles of 38.0° (layer 1) and 6.6° (layer 3), respectively. Layers 2 and 4 (Figure 2e,g) are symmetry‐related by a 2_1_ screw axis running along the *b*‐direction. They consist of yttrium atoms and pentazolate rings, which are nearly aligned with the *bc* plane.

**Figure 2:**
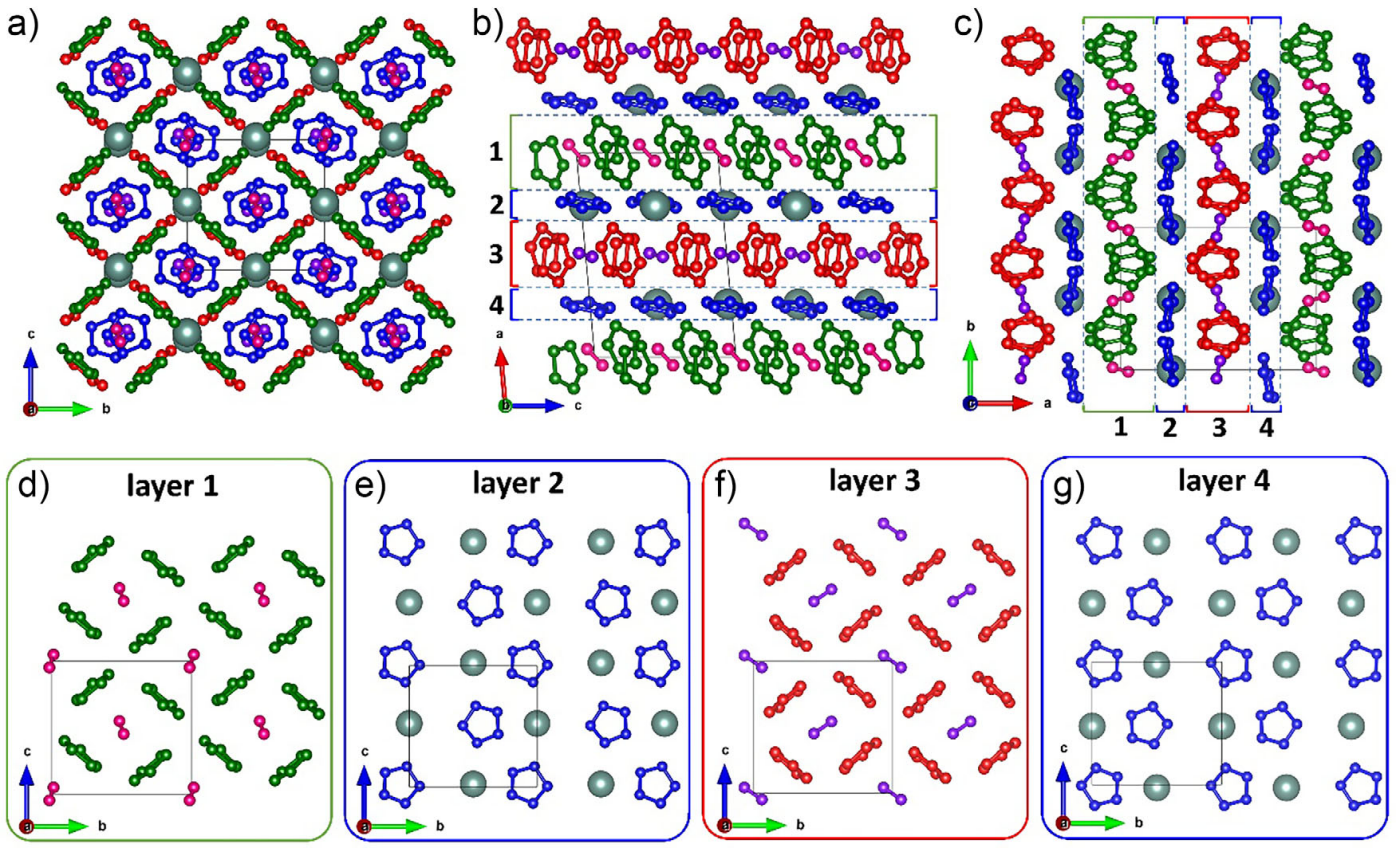
Crystal structure of Y(N_5_)_3_·N_2_ at 125(2) GPa. View along a) *a* direction, b) *b* direction, and c) *c*‐direction. d)–g) four layers representing the stacking along *a* direction. Greenish big balls correspond to yttrium atoms; small red, green, and blue balls correspond to nitrogen atoms of three distinct pentazolate rings; and small purple and pink balls correspond to nitrogen atoms of two distinct nitrogen dimers. Grey thin lines outline the unit cell.

The average bond length in pentazolate rings in Y(N_5_)_3_·N_2_ at 125 GPa is 1.269(4) Å (Figure 3a), which is reasonably smaller than the bond lengths of pentazolates at ambient conditions (1.31–1.33 Å)^[^ ^1^, ^2^, ^3^, ^4^, ^5^, ^6^, ^7^ ^]^ and at moderate pressures (e.g., 1.29–1.30 Å in NaN_5_ at 53 GPa)^[^ ^9^ ^]^ The nitrogen─nitrogen bond lengths in the dimers are 1.08(3) and 1.06(3) Å, consistent with a triple N≡N bond, indicating that the dimers are non‐charged. Yttrium atoms are twelve‐fold coordinated (coordination number CN = 12) by N atoms from nine pentazolate rings (Figure 3b). The distances within the YN_12_ polyhedra range from 2.221(11) to 2.344(7) Å. These polyhedra do not share faces, edges, or vertices and are connected into a 3D framework solely by pentazolate rings. This framework contains two types of pores, each accommodating a nitrogen dimer surrounded by six pentazolates (Figure 3c,d).

**Figure 3:**
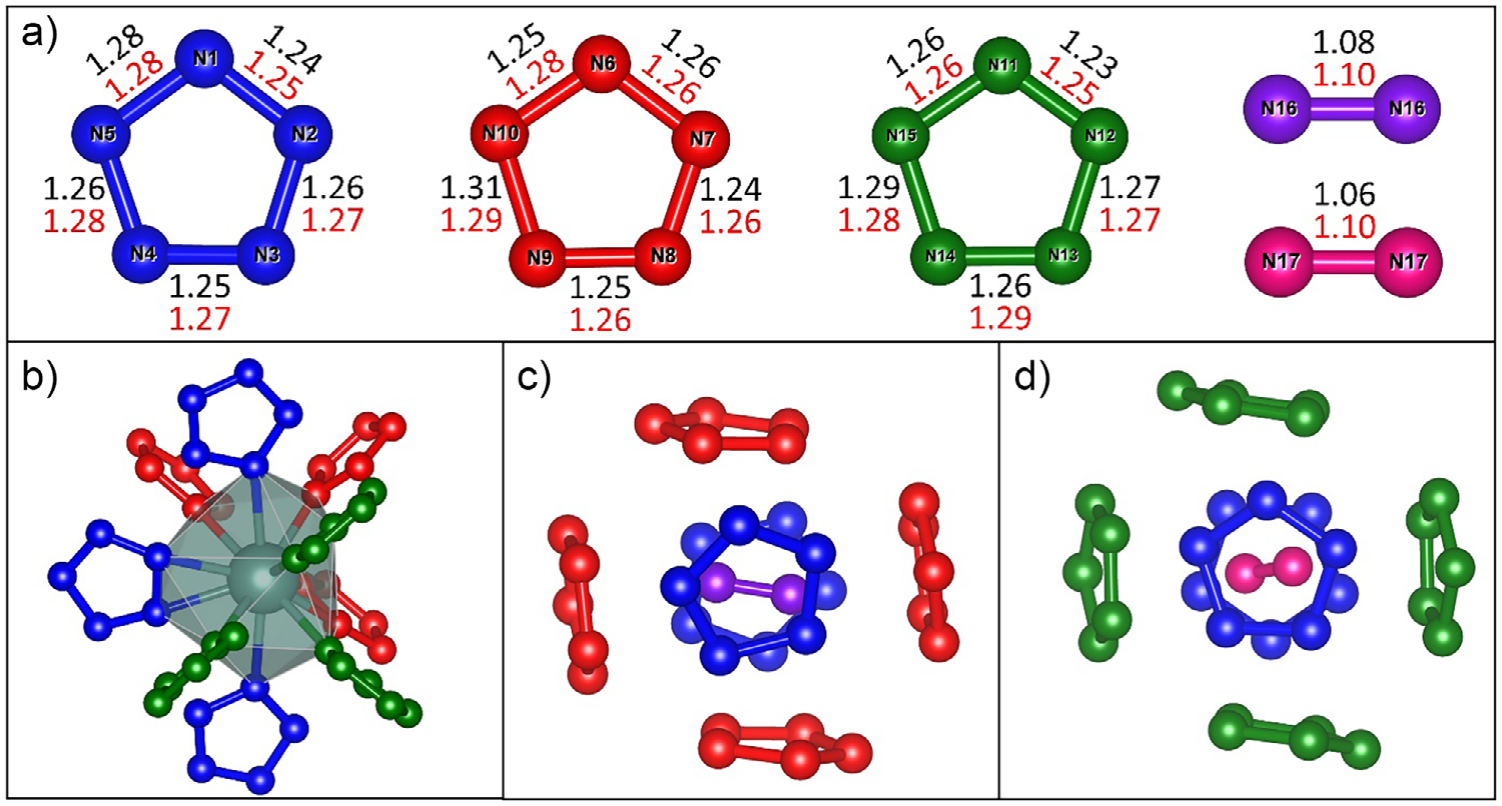
Geometry of pentazolate rings and nitrogen dimers and their arrangement in Y(N_5_)_3_·N_2_ at 125(2) GPa. a) Bond lengths in pentazolate rings and nitrogen dimers. The values obtained from experiments are shown in black, while those obtained from the DFT calculations are shown in red. b) The coordination environment of yttrium atoms. c) The environment of the N16–N16 dimer d) The environment of the N17–N17 dimer. The greenish big balls represent yttrium atoms; red, green, and blue balls represent nitrogen atoms from three distinct pentazolate rings; and the purple and pink balls correspond to nitrogen atoms from two distinct nitrogen dimers.

The compound Y(N_5_)_3_·N_2_ can be classified as a nitrogen‐inclusion metal‐pentazolate framework and represents only the second known example of this class, following NaN_5_⋅N_2_,^[^ ^9^ ^]^ although other high‐pressure nitrogen‐inclusion compounds, such as ReN_8_·xN_2_, WN_8_·N_2_, Os_5_N_28_·3N_2_, Hf_4_N_20_·N_2_, and K_9_N_56_, are known.^[^ ^10^, ^20^, ^21^ ^]^ Notably, Y(N_5_)_3_·N_2_ is the first experimentally observed compound to feature a 3:1 pentazolate‐to‐metal ratio.

In the analysis of complex metal‐organic compounds, polyatomic organic ligands are commonly considered as building units of the crystal structures. This approach allows complicated structures to be described using simple molecular components or groups, facilitating the construction of giant structures with esthetically appealing geometries.^[^ ^22^ ^]^ Similar considerations are also employed in ToposPro to determine the topology of various classes of chemical compounds, such as molecular crystals, inorganic ionic compounds, coordination polymers, and supramolecular ensembles.^[^ ^23^, ^24^ ^]^ By considering pentazolate rings and nitrogen dimers as structural units and representing them by their geometrical centers, one can see a distorted perovskite motif in the crystal structure of Y(N_5_)_3_·N_2_: the centers of pentazolate anions and nitrogen molecules form a distorted octahedral ReO_3_‐type framework, with distorted cuboctahedral voids occupied by yttrium atoms (Figure 4). Inspired by this finding, we analyzed the structures of all currently synthesized inorganic solvent‐free pentazolate compounds using a similar centroid‐based structure typification approach, replacing pentazolate anions and additional structural units with their geometrical centers (see Supplementary Discussion). Our analysis reveals that the structure topology of these compounds is very diverse and comprises the motifs of both previously known, and to date, unknown structure types (see Supplementary Discussion).

**Figure 4:**
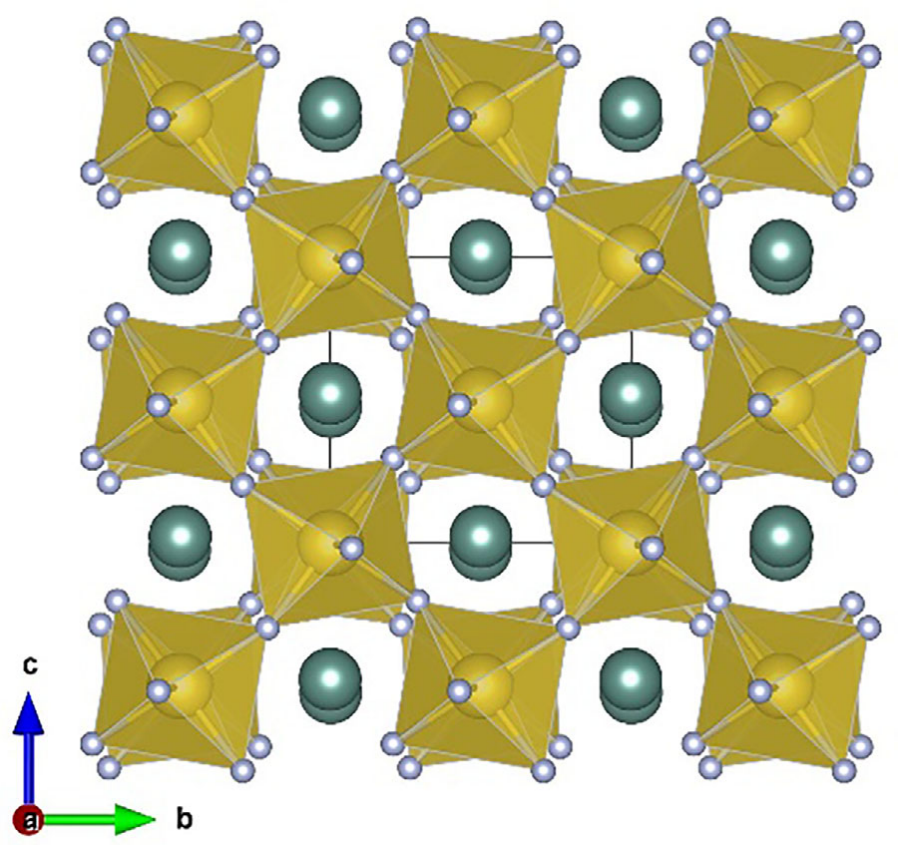
The simplified crystal structure of Y(N_5_)_3_·N_2_, where pentazolate rings and nitrogen dimers are replaced with their centers of mass viewed along the *a* axis, revealing a distorted perovskite motif. Greenish big balls correspond to yttrium atoms; grey balls correspond to the centers of pentazolate rings; yellow balls correspond to the centers of nitrogen dimers. Black, thin lines outline the unit cell.

After the Y(N_5_)_3_·N_2_ synthesis at 125(2) GPa, we attempted to decompress the sample down to ambient conditions to investigate its behavior upon the decompression, the equation of state, and recoverability. Due to technical limitations, only four pressure points down to 57(2) GPa were successfully collected, and then it was no longer possible to continue the experiment (Table S2). XRD data confirm the existence of Y(N_5_)_3_·N_2_ down to 57(2) GPa (Figure S2). This experiment does not provide conclusions about the recoverability of Y(N_5_)_3_·N_2_; however, due to the presence of weakly‐bonded N_2_ dimers in the crystal structure, decomposition is expected when the pressure approaches ambient conditions.

For cross‐validation of the Y(N_5_)_3_·N_2_ crystal structure and to get a deeper insight into the stability and physical properties of the novel compound, we performed density functional theory (DFT) calculations (see Methods section in Supporting Information). We carried out variable cell structural relaxation for Y(N_5_)_3_·N_2_ at 125 GPa and found that the relaxed unit cell volume and unit cell parameters closely reproduce the corresponding experimental values (Table S3); the calculated bond lengths are also in good agreement with the experimental data (Figure 3).

The calculated electron localization function for Y(N_5_)_3_·N_2_ demonstrates a strong covalent bonding between nitrogen atoms within the pentazolates (Figure 5a,b) and within N_2_ dimers (Figure 5c). The attractor associated with the N─N bonds located halfway between the nitrogen atoms is larger in the case of N_2_ dimers than within N─N bonds in pentazolates, demonstrating a higher bond order of the former. Each nitrogen atom of pentazolate anions has a lone electron pair protruding in the plane of the ring. Yttrium atoms are coordinated by pentazolate anions via one or two lone pairs (Figure 5a,b). Nitrogen dimers are surrounded by six pentazolate rings (six distinct charge density pieces around each dimer in Figure 5c), and there is no covalent interaction between them.

**Figure 5:**
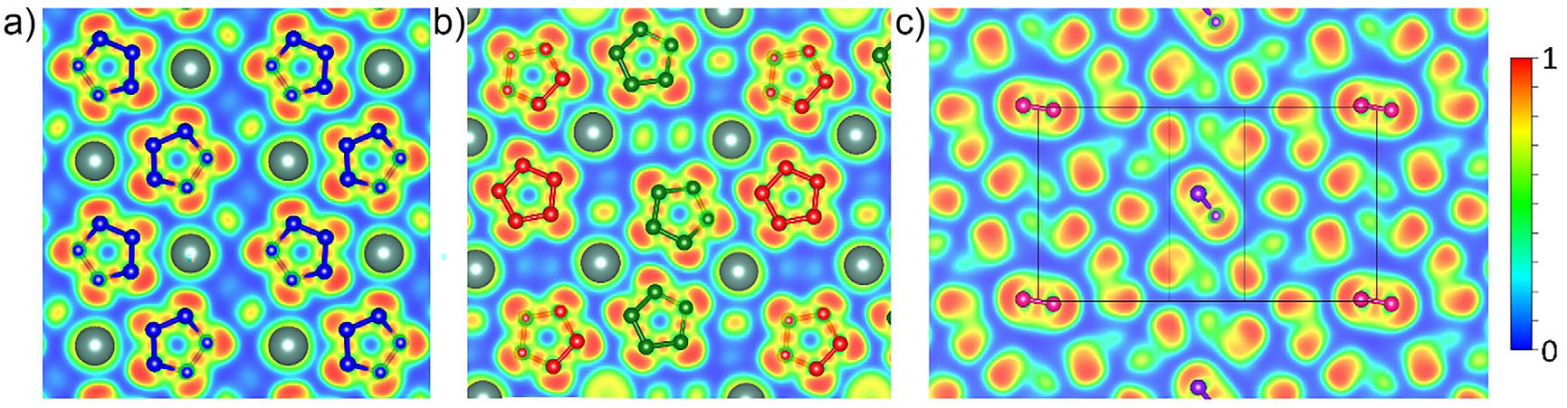
Cross sections of the electron localization function calculated for Y(N_5_)_3_·N_2_ at 125 GPa in a) the (4 0 0) plane, b) the (0 −2 2) plane, and c) the (1 0 1) plane.

The phonon dispersion relations calculated in the harmonic approximation show that Y(N_5_)_3_·N_2_ is dynamically stable at the synthesis pressure of 125 GPa (Figure 6a). The remarkable feature of the phonon dispersions is the separated high‐frequency optic modes (at 75–78 THz) that correspond to the vibrations of non‐charged triple‐bonded nitrogen dimers. The same characteristic phonon density of states feature is demonstrated by other nitrogen‐inclusion polynitrides, i.e., ReN_8_·xN_2_,^[^ ^20^ ^]^ WN_8_·N_2_, Hf_4_N_20_·N_2_, and Os_5_N_28_·3N_2_.^[^ ^21^ ^]^

**Figure 6:**
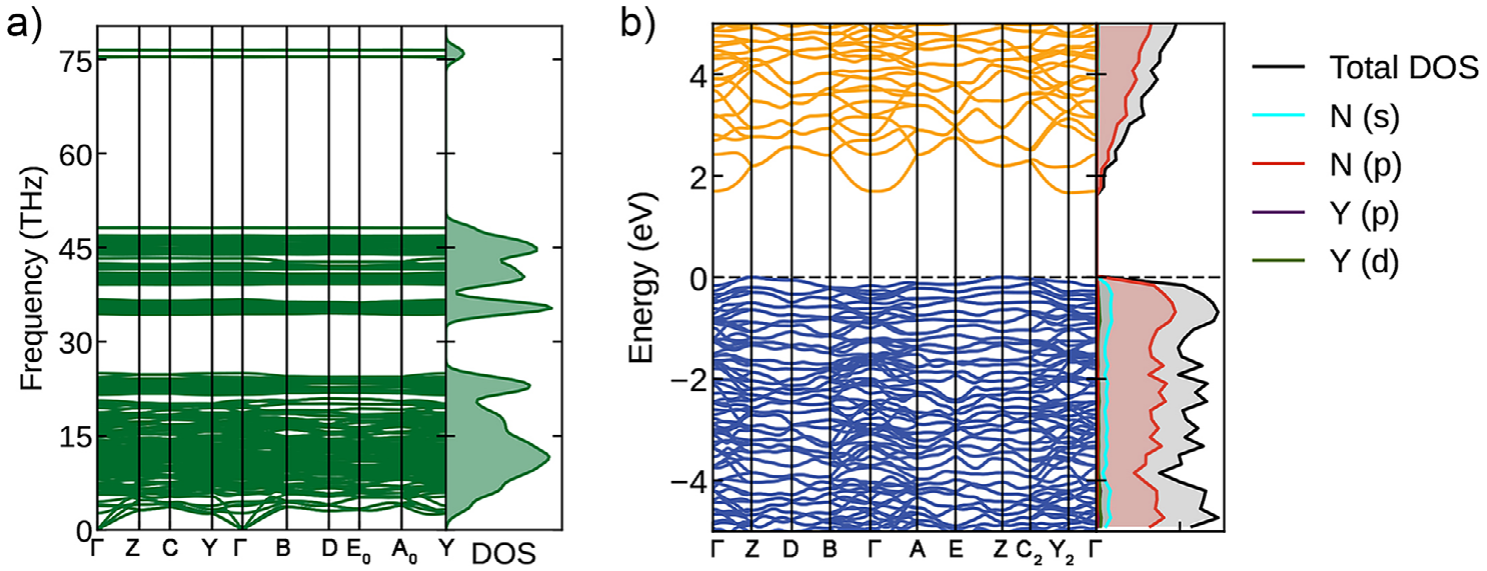
Results of DFT calculations for Y(N_5_)_3_·N_2_ at 125 GPa. a) Phonon dispersion curves along high‐symmetry directions in the Brillouin zone and phonon density of states. b) Electron band structure and electron density of states calculated using PBE; energy is shifted with respect to the highest occupied state.

The computed electron density of states shows that Y(N_5_)_3_·N_2_ is a semiconductor with a band gap of 1.61 eV (from PBE) at 125 GPa (Figure 5b). Nitrogen *p*‐states dominate both conduction and valence bands. In the di‐ and poly‐nitrides, it is a very common phenomenon, and usually, the filling of the molecular orbitals of the nitrogen units dictates the metal/semiconductor properties of the compound. When nitrogen units have partially filled π* orbitals, one can expect the anion‐driven metallicity of the compound, which is very common in di‐ (LiN_2_, Li_2_N_2_, NaN_2_, Na_3_(N_2_)_4_, K_3_(N_2_)_4_, CaN_2_, Ca_3_(N_2_)_4_, SrN_2_, Sr_3_(N_2_)_4_, BaN_2_, Ba(N_2_)_3_, CoN_2_, RuN_2_, Y_5_N_14_),^[^ ^8^, ^17^, ^25^, ^26^, ^27^, ^28^, ^29^, ^30^ ^]^ oligo‐ (TaN_4_, Sc_2_N_6_, Sc_2_N_8_, YN_6_)^[^ ^18^, ^31^, ^32^ ^]^ and poly‐nitrides (tr‐BeN_4_, FeN_4_, α‐ZnN_4_, β‐ZnN_4_, TaN_4_, ReN_8_·xN_2_, WN_8_·N_2_, Os_5_N_28_·3N_2_, Hf_4_N_20_·N_2_, Hf_2_N_11_, Y_2_N_11_,).^[^ ^18^, ^20^, ^21^, ^31^, ^33^, ^34^, ^35^ ^]^ While the absence of partial orbital occupation results in the semiconductor properties of the corresponding material. This is the case of (1) single‐bonded anions; those π* orbitals are fully occupied: pernitrides N_2_^4‐^ (PtN_2_),^[^ ^36^ ^]^ single‐bonded polynitrogen chains (TaN_5_),^[^ ^31^ ^]^ single‐bonded polynitrogen layers (ScN_5_ and BeN_4_)^[^ ^32^, ^33^ ^]^ or (2) aromatic pentazolates N_5_^−^, which also do not have partially occupied molecular orbitals.^[^ ^37^ ^]^ The semiconductor properties of Y(N_5_)_3_·N_2_ also follow this trend, since both structure‐forming nitrogen units of Y(N_5_)_3_·N_2_—aromatic pentazolates and non‐charged nitrogen dimers—do not have partially occupied states.

To trace the structural behavior during the pressure release and to get the equation of state of Y(N_5_)_3_·N_2_, the full variable‐cell structure relaxations of Y(N_5_)_3_·N_2_ were performed with 10 GPa pressure steps in the range of 0–130 GPa. PBE calculations suggest that the initial Y(N_5_)_3_·N_2_ structure is stable at least down to 40 GPa (Figures S3–S5). At 30 GPa, the significant rotation of nitrogen dimers is observed (Figure S4), although the structure remains dynamically stable (Figure S6). The recognized mobility of nitrogen dimers below 40 GPa might result either in (1) an isostructural phase transition associated with the dimers' rotation or (2) decomposition of Y(N_5_)_3_·N_2_ with nitrogen being released. At ambient conditions, Y(N_5_)_3_·N_2_ is no longer stable, which one might have expected considering the presence of neutral and loosely bonded N_2_ dimers. The volume‐pressure dependence of DFT‐relaxed structures of Y(N_5_)_3_·N_2_ in the pressure range of 40–130 GPa was fitted with a 2^nd^‐order Birch–Murnaghan equation of state (Figure S7), leading to the bulk modulus of K_0_(Y(N_5_)_3_·N_2_) = 80.5(11) GPa. The obtained bulk moduli are lower than the bulk moduli of other experimentally known yttrium nitrides, and the bulk modulus decreases with the decrease of yttrium content: K_0_(YN) = 151.0 GPa^[^ ^16^ ^]^ > K_0_(Y_5_N_14_) = 137.0 GPa^[^ ^17^ ^]^ > K_0_(Y_2_N_11_) = 115.7 GPa^[^ ^18^ ^]^ > K_0_(YN_6_) = 92.6 GPa^[^ ^18^ ^]^ > K_0_(Y(N_5_)_3_·N_2_) = 80.5 GPa.

To estimate the thermodynamic stability of Y(N_5_)_3_·N_2_ in its existence domain, we calculated static enthalpy convex hulls at 125, 100, and 50 GPa (Figure S8). Only experimentally observed Y‐N compounds, namely YN, Y_5_N_14_, Y_2_N_11_, and YN_6_, were considered. At 125 GPa, Y(N_5_)_3_·N_2_ lies 113 meV per atom above the convex hull; this value is significantly smaller than k_B_T at synthesis temperature (3000 K, 258 meV). Upon decreasing pressure, Y(N_5_)_3_·N_2_ becomes closer to the convex hull, and at 100 GPa it is only slightly (43 meV/atom) above the convex hull. At 50 GPa, Y(N_5_)_3_·N_2_ lies on the convex hull.

It was shown that a larger amount of energetic nitrogen oligomers per counterion leads to higher energy density.^[^ ^12^ ^]^ Therefore, due to the high pentazolate‐to‐metal ratio, the Y(N_5_)_3_·N_2_ compound is expected to be a promising HEDM. However, since, according to DFT, the Y(N_5_)_3_·N_2_ phase is no longer stable at ambient conditions, the most reliable energy densities—at ambient pressure—cannot be calculated.

To summarize, in this study, a novel binary compound Y(N_5_)_3_·N_2_ with an exceptionally high nitrogen‐to‐metal ratio of 17:1 was synthesized from Y and N_2_ by laser heating at 125 GPa and 3000 K. The crystal structure of Y(N_5_)_3_·N_2_ represents a metal‐pentazolate framework with voids filled by nitrogen molecules. DFT calculations support experimental findings and show that Y(N_5_)_3_·N_2_ is a semiconductor. Under decompression, Y(N_5_)_3_·N_2_ was preserved down to at least 57(2) GPa, and DFT calculations predict its existence down to approximately 40 GPa. The primary reason for its instability at ambient conditions is the presence of weakly‐bonded N_2_ molecule inclusions within the structure. Because of instability at ambient pressure, Y(N_5_)_3_·N_2_ cannot be considered as HEDM under ambient conditions. Nonetheless, the stabilization of three pentazolate anions per metal cation demonstrated in this study suggests that nitrogen‐inclusion‐free metal‐pentazolate frameworks with a 3:1 (or even higher) pentazolate‐to‐metal ratio could potentially be stabilized at high pressures and recovered to ambient conditions. Such frameworks would hold significant promise for high‐energy‐density applications.

Additionally, we proposed a centroid‐based structure typification for solvent‐free inorganic pentazolates. We demonstrated that the relatively large N^5−^ anion, often combined with other nitrogen units, is inclined to form various structural motifs, some of which are already known. Notably, in Y(N_5_)_3_·N_2,_ the centers of mass of pentazolate anions and nitrogen molecules create a distorted ReO_3_‐type framework of octahedra, with distorted cuboctahedral voids occupied by yttrium atoms, resulting in a perovskite‐like motif. Considering the wide range of intriguing properties exhibited by materials with perovskite topology, this finding may pave the way for the discovery of additional pentazolates with similar structures and their potential applications in the future.

## Supporting Information

The authors have cited additional references within the Supporting Information.^[^ ^38^, ^39^, ^40^, ^41^, ^42^, ^43^, ^44^, ^45^, ^46^, ^47^, ^48^, ^49^, ^50^, ^51^, ^52^, ^53^, ^54^, ^55^, ^56^, ^57^ ^]^

## Conflict of Interests

The authors declare no conflict of interest.

## Supporting information



Supporting Information S1

Supporting Information S2

## Data Availability

The data that support the findings of this study are available in the Supporting Information of this article.
