# Supplementary material for: High‐Pressure Synthesis of Nitrogen‐Rich Y(N5)3·N2 Pentazolate with Perovskite Topology
Source: Angew Chem Int Ed Engl. 2025 Jun 16;64(31):e202506334. doi: 10.1002/anie.202506334 (PMC12304802; doi:10.1002/anie.202506334)
Supplement: Supplementary file 1 — Supporting Information S1 [file ANIE-64-e202506334-s002.pdf]

Supporting information

High-pressure synthesis of nitrogen-rich $Y(N_5)_3{\cdot}N_2$ pentazolate with perovskite topology

Andrey Aslandukov[1,2,3*], Yuqing Yin[2,4], Maxim Bykov[3], Alena Aslandukova[1,3], Fariia I. Akbar[1,3], Eleanor Lawrence Bright[5], Igor A. Abrikosov[4], Natalia Dubrovinskaia[2,4], Leonid Dubrovinsky[1*]

[1] Bavarian Research Institute of Experimental Geochemistry and Geophysics (BGI), University of Bayreuth, 95440 Bayreuth, Germany

[2] Material Physics and Technology at Extreme Conditions, Laboratory of Crystallography, University of Bayreuth, 95440 Bayreuth, Germany

[3] Goethe University Frankfurt, Institute of Inorganic and Analytical Chemistry, 60438 Frankfurt, Germany

[4] Department of Physics, Chemistry and Biology (IFM), Linköping University, SE-581 83, Linköping, Sweden

[5] European Synchrotron Radiation Facility, 38000 Grenoble, France

Table of Contents

Methods..2
Supplementary discussion. Structure typification of inorganic solvent-free pentazolates........4
Supplementary Tables 6
Supplementary Figures 8
References..12

Methods

Sample preparation. The BX90-type large X-ray aperture DAC[1] equipped with Boehler-Almax type diamonds[2] (culet diameter of 120 µm) was used in the experiment. The sample chambers were formed by pre-indenting of rhenium gaskets to 18 µm thickness and laser-drilling a hole of 60 µm in diameter in the center of the indentation. A piece of yttrium (99.9%, Sigma Aldrich) was placed in a sample chamber, then molecular nitrogen was loaded using a high-pressure gas loading system (1300 bars).[3] The samples were compressed to target pressure of 125(2) GPa and laser-heated (λ = 1064 nm) up to 3000(300) K using a home-made double-sided laser-heating system at BGI equipped with two YAG lasers (λ = 1064 nm) and the IsoPlane SCT 320 spectrometer with a 1024×2560 PI-MAX 4 camera for the collection of thermal emission spectra from the heated spot.[4] The temperature during the laser heating was determined by fitting of sample's thermal emission spectra to the grey body approximation of Planck's radiation function in a given wavelength range (570–830 nm). The pressure in the DACs was determined using the Raman signal from the diamond anvils[5] and monitored additionally by X-ray diffraction of the Re gasket edge using the rhenium equation of state.[6]

X-ray diffraction. The X-ray diffraction studies were done at the ID11 beamline (λ = 0.2846 Å) of the Extreme Brilliant Source European Synchrotron Radiation Facility (EBS-ESRF). X-ray beam was focused down to 0.75 × 0.75 µm^2 and data was collected with Eiger2X CdTe 4M hybrid photon counting pixel detector. In order to determine the position of the polycrystalline sample on which the single-crystal X-ray diffraction acquisition is obtained, a full X-ray diffraction mapping of the pressure chamber was achieved. The sample position displaying the most and the strongest single-crystal reflections belonging to the phase of interest was chosen for the collection of single-crystal data, collected in step-scans of 0.5° from −36° to +36°. The CrysAlis^{Pro} software package[7] was used for the analysis of the single-crystal XRD data (peak hunting, indexing, data integration, frame scaling, and absorption correction). To calibrate an instrumental model in the CrysAlis^{Pro} software, i.e., the sample-to-detector distance, detector's origin, offsets of the goniometer angles, and rotation of both the X-ray beam and detector around the instrument axis, we used a single crystal of orthoenstatite

[$(Mg_{1.93}Fe_{0.06})(Si_{1.93},Al_{0.06})O_6$, *Pbca* space group, a = 8.8117(2) Å, b = 5.18320(10) Å, and c = 18.2391(3) Å]. The DAFi program was used for the search of reflection's groups belonging to the individual single crystal domains.[8] Using the OLEX2 software package,[9] the structures were solved with the ShelXT structure solution program[10] using intrinsic phasing and refined with the ShelXL[11] refinement package using least-squares minimization. Crystal structure visualization was made with the VESTA software.[12]

Theoretical Calculations. First-principles calculations were performed using the framework of density functional theory (DFT) as implemented in the Vienna Ab initio Simulation Package (VASP).[13] The Projector-Augmented-Wave (PAW) method[14] was used to expand the electronic wave functions in a plane wave basis. The Generalized Gradient Approximation (GGA) functional is used for calculating the exchange-correlation energies, as proposed by Perdew–Burke–Ernzerhof (PBE).[15] The "Y_sv" and "N" PAW potentials with the following valence configurations of 4s4p5s4d for Y, and 2*s*2*p* for N were used. In geometry optimization, we used a Gamma centered 5×7×7 k-mesh, and the plane-wave kinetic energy cutoff was set to 1000 eV, with which total energies are converged to better than 1 meV/atom. The electronic convergence criterion was set to $\Delta E = 10^{-8}$ eV, this minimized the interatomic forces to $F_{atom} < 10^{-3}$ eV/Å. To increase the accuracy of ground-state electron density and density of states (DOS), a denser Gamma centered k-mesh of 9×13×13 and the tetrahedron smearing method with Blöchl corrections (ISMEAR = -5) were used. The finite displacement method, as implemented in PHONOPY, was used to calculate harmonic phonon frequencies and phonon band structures. A supercell size of 2×2×2 were used with k-mesh size of 2×2×2 for the harmonic phonon calculations at 0 K and displacement amplitudes were of 0.01 Å.

Supplementary discussion. Structure typification of inorganic solvent-free pentazolates.

Here we proposed a centroid-based structure typification of up-to-date experimentally synthesized inorganic solvent-free pentazolates (Table SD1). This typification implies polyhedral description and determination of structure type of simplified pentazolates structures, where N_5 rings (and, if any, other several-atomic units, e.g. N_2, N_3^-, NH_4^+) are replaced by their center of mass (Table SD2, Fig SD1).

Table SD1. Experimentally synthesized inorganic solvent-free pentazolate compounds

Compound*	Spacegroup	Method**	CCDC code	Reference
HP-LiN_5	*P*2$_1$/*m*	PXRD	2185696	[16]
HP-NaN_5	*Cm*	PXRD	2186589	[17]
HP-NaN_5	*Pmn*2$_1$	SCXRD	2051780	[18]
HP-CsN_5	*P*-1	PXRD+DFT	ICSD126030	[19]
KN_5	*Pnma*	SCXRD	1885286	[20]
NH_4N_5	*Pcca*	SCXRD	1887029	[20]
LiN_5 (LPF)	*Pm*-3*n*	SCXRD	2097922	[21]
AgN_5	*Fddd*	SCXRD	1816845	[22]
HP-$NaN_5\cdot N_2$	*P*2$_1$/*n*	SCXRD	2051781	[18]
CuN_5N_3	*Ima*2	SCXRD	1816844	[22]
HP-$Y(N_5)_3\cdot N_2$	*P*2$_1$/*c*	SCXRD	2407655	Present study

*HP label indicates high-pressure phases

**Method used for crystal structure determination: SCXRD – single-crystal X-ray diffraction; PXRD – powder X-ray diffraction; PXRD+DFT – structure found as a result of ab initio structure search, that fits a powder XRD pattern

Table SD2. Centroid-based structure typification of experimentally synthesized inorganic solvent-free pentazolate compounds

Compound	Simplified compound	Polyhedra	Structure type of the simplified compound
HP-LiN_5	AB	Distorted octahedra AB_6	distorted NiAs
HP-NaN_5	AB	Distorted cubes AB_8	distorted CsCl
HP-NaN_5	AB	Distorted octahedra AB_6	distorted NaCl
HP-CsN_5	AB	Irregular AB_7 and AB_8	unknown
KN_5	AB	Distorted octahedra AB_6	highly-distorted NaCl
NH_4N_5	AB	Distorted cubes AB_8	distorted CsCl
LiN_5 (LPF)	AB	AB_4 squares and AB_5 pyramids	unknown
AgN_5	AB	Distorted tetrahedra AB_4	distorted anti-PtS
HP-$NaN_5\cdot N_2$	ABC	Trigonal prisms AB_6 + C in the voids	unknown
CuN_5N_3	ABC	Distorted octahedra AB_4C_2	unknown
HP-$Y(N_2)(N_5)_3$	ABX_3	Distorted octahedra BX_6	distorted perovskite

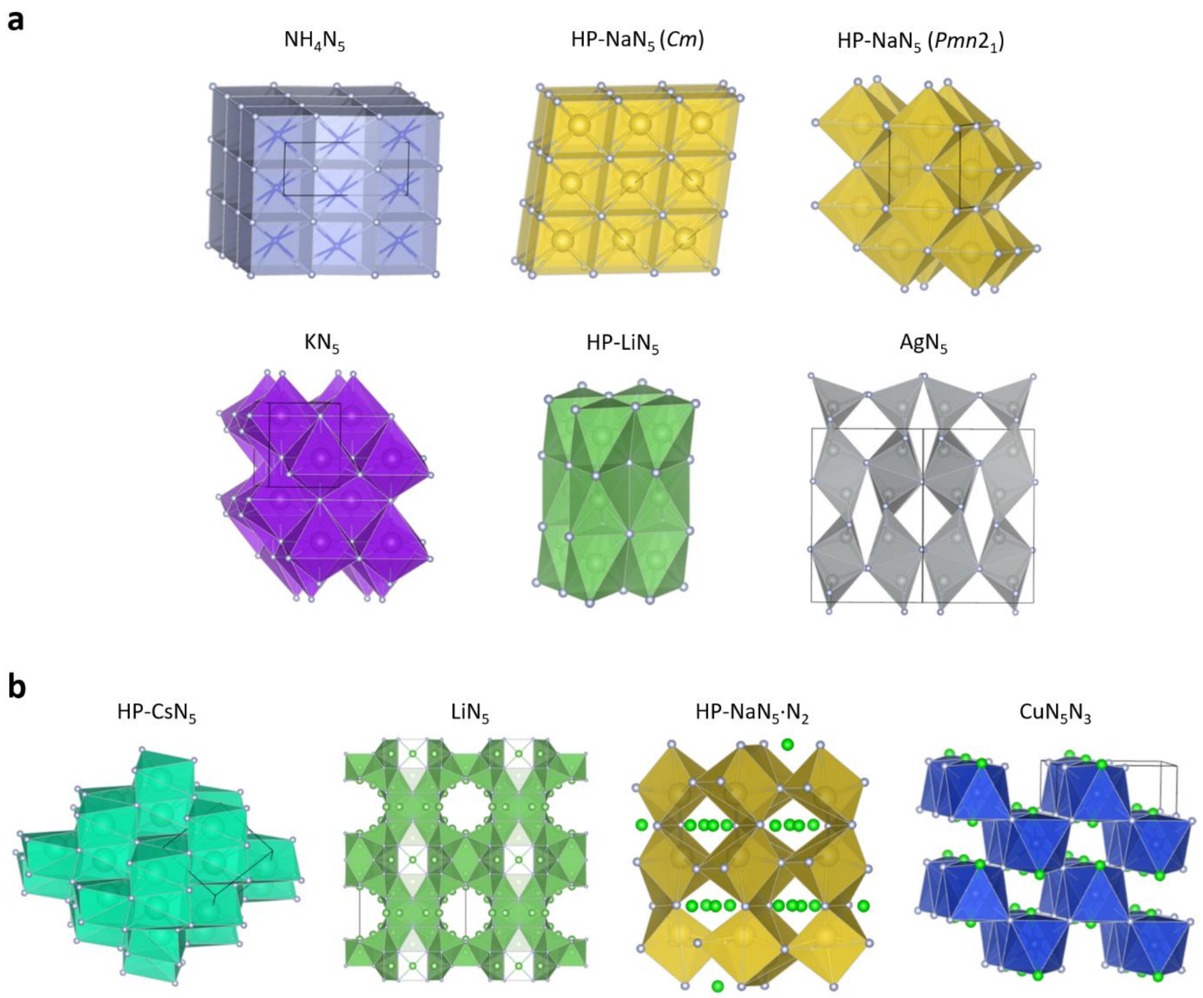

Figure SD1. The simplified crystal structures of inorganic solvent-free pentazolates, where rings (and any additional polyatomic units, such as N_2, N_3^-, NH_4^+) are replaced by their center of mass. Grey balls correspond to the centers of pentazolate rings. Green balls in HP-NaN_5·N_2 and CuN_5N_3 correspond to the centers of N_2 and N_3^- units. Other colors correspond to metal atoms (or centers of NH_4^+ cations in NH_4N_5) yellow balls correspond to the centers of nitrogen dimers. (a) Compounds with assigned known structure types and (b) compounds with yet unknown structure types.

Analysis shows that 7 out of 11 compounds have centroid-based simplified crystal structures with known distorted structure types (Table SD2, Fig. SD1a, Fig. 4). While four compounds exhibit unique structure motifs (Table SD2, Fig. SD1b).

Supplementary Tables

Table S1. Structure refinement details of $Y(N_5)_3 \cdot N_2$ at 125 GPa. The full crystallographic data was deposited to the ICSD under the deposition number CSD 2407655. The A and B alerts revealed by CheckCIF are listed and explained after the table.

Chemical formula					$Y(N_5)_3 \cdot N_2$
Temperature (K)					293
Pressure (GPa)					125(2)
**Crystal data**					
Mr					327.08
ρ (g/cm^3)					5.092
Crystal system, space group					monoclinic, $P2_1/c$
a (Å)					9.6169(12)
b (Å)					6.932(5)
c (Å)					6.6089(11)
β (°)					95.134(14)
V (Å^3)					438.8(3)
Z					4
Radiation type					X-ray, λ = 0.2846 Å
μ (mm^{-1})					4.951
**Data collection**					
No. of measured, independent and observed [I > 2σ(I)] reflections					1447/ 756/ 467
R_{int}					5.80%
(sin θ/λ)$_{max}$ (Å^{-1})					0.768
**Refinement**					
R[F^2 > 4σ(F^2)], wR(F^2), GOF					4.89%, 11.02%, 1.132
data/parameters ratio					756/78
$\Delta\rho_{max}$, $\Delta\rho_{min}$ (e Å^{-3})					1.441, -0.963
**Atomic positions and equivalent isotropic (or isotropic) ADPs**					
Atom	Wyckoff site	Fractional atomic coordinates			U_{iso} or U_{eq} (Å^2)
		x	y	z	
Y1	4*e*	0.25989(6)	0.0008(3)	0.47665(10)	U_{eq} = 0.0054(3)
N1	4*e*	0.2680(6)	0.020(3)	0.8316(10)	U_{iso} = 0.0086(13)
N2	4*e*	0.2443(7)	0.189(2)	0.8812(11)	U_{iso} = 0.0067(14)
N3	4*e*	0.2182(7)	0.196(2)	0.0651(11)	U_{iso} = 0.0051(13)
N4	4*e*	0.2421(6)	0.031(3)	0.1358(10)	U_{iso} = 0.0084(13)
N5	4*e*	0.2686(7)	0.581(2)	0.4938(11)	U_{iso} = 0.0071(14)
N6	4*e*	0.4223(7)	0.186(3)	0.6516(12)	U_{iso} = 0.0073(15)
N7	4*e*	0.4611(7)	0.617(2)	0.8829(12)	U_{iso} = 0.0079(14)
N8	4*e*	0.3677(7)	0.714(2)	0.7898(11)	U_{iso} = 0.0092(15)
N9	4*e*	0.4216(8)	0.659(3)	0.1882(12)	U_{iso} = 0.0090(17)
N10	4*e*	0.4424(7)	0.180(2)	0.2763(11)	U_{iso} = 0.0057(14)
N11	4*e*	0.1530(7)	0.705(2)	0.1684(12)	U_{iso} = 0.0086(14)
N12	4*e*	0.0611(7)	0.593(2)	0.1012(12)	U_{iso} = 0.0102(15)
N13	4*e*	0.0540(7)	0.145(2)	0.3339(11)	U_{iso} = 0.0066(14)
N14	4*e*	0.0288(7)	0.216(2)	0.7144(11)	U_{iso} = 0.0070(14)
N15	4*e*	0.1040(7)	0.683(2)	0.7937(11)	U_{iso} = 0.0085(15)
N16	4*e*	0.4910(7)	0.563(2)	0.5457(10)	U_{iso} = 0.0084(15)
N17	4*e*	0.0398(6)	0.515(3)	0.4513(10)	U_{iso} = 0.0112(13)

CheckCIF alerts:

PLAT911_ALERT_3_B Missing FCF Refl Between Thmin & STh/L= 0.600 380 Report

Author Response: This measurement was performed at high pressure which, due to the high pressure apparatus, limits the theta range. Indeed, the diamond anvil cell metallic body typically shadows more than 60% of the reflections.

Table S2. The lattice parameters of $Y(N_5)_3{\cdot}N_2$ at different pressures.

Pressure, GPa*	a, Å	b, Å	c, Å	β, °	Volume, Å^3
125(2)	9.6169(12)	6.932(5)	6.6089(11)	95.134(14)	438.8(3)
111(2)	9.6715(14)	6.9905(15)	6.6889(11)	95.528(14)	450.13(14)
98(2)	9.799(2)	7.0669(12)	6.7356(12)	95.486(17)	464.29(15)
78(2)	9.9876(14)	7.1953(12)	6.8364(11)	96.004(13)	488.60(13)
57(2)	10.2586(17)	7.377(3)	7.0138(15)	96.52(2)	527.3(2)

*After the compound's synthesis at 125(2) GPa, the DAC was decompressed from that pressure down to 57(2) GPa. At the next pressure point the nitrogen pressure transmitting medium went out through microcracks in the diamond resulting in a diamond breaking and the complete gasket closure, with the pressure dropping to 1 atm. The sample could not be found afterwards.

Table S3. Experimentally determined crystallographic data for $Y(N_5)_3{\cdot}N_2$ phase at 125 GPa in comparison with the corresponding DFT-relaxed structure. Note that pressure was fixed in theoretical simulations, while volumes of the unit cells, lattice parameters and equilibrium state parameters were calculated.

	**Exp.**	**Calc.**
**Space group**	*P*2$_1$/*c*	*P*2$_1$/*c*
**Volume**	438.8(3) Å^3	439.6 Å^3
**Lattice parameters**	a = 9.6169(12) Å<br>b = 6.932(5) Å<br>c = 6.6089(11) Å<br>β = 95.134(14)°	a = 9.6103 Å<br>b = 6.9206 Å<br>c = 6.6365 Å<br>β = 95.160°

Supplementary Figures

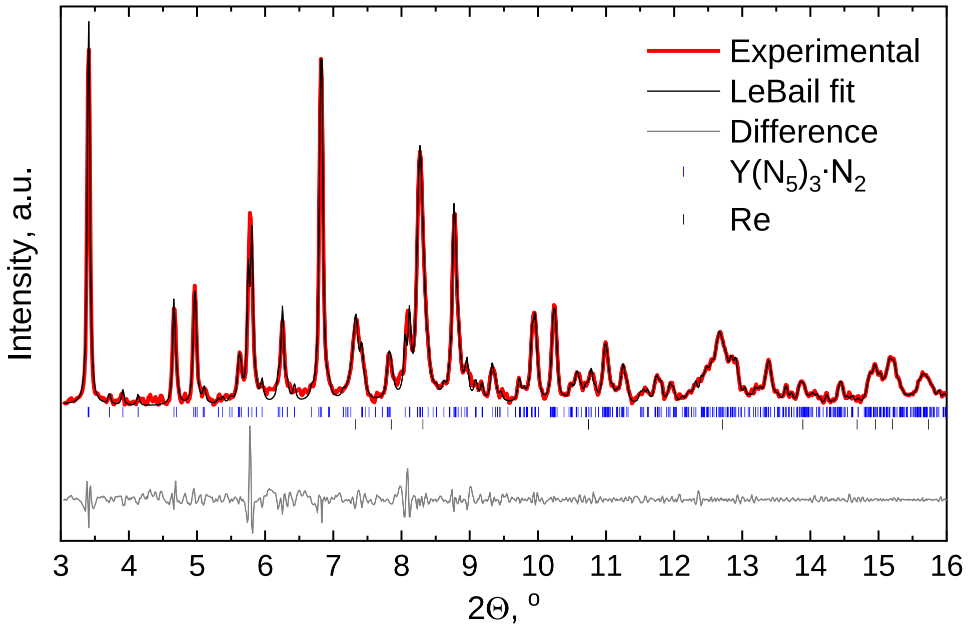

Fig. S1. Integrated powder XRD pattern of the sample at 125 GPa (λ = 0.2846 Å) and its Le Bail fit using unit cell parameters of $Y(N_5)_3{\cdot}N_2$ obtained from the single-crystal XRD data. All reflections can be explained by $Y(N_5)_3{\cdot}N_2$ phase and Re from the gasket.

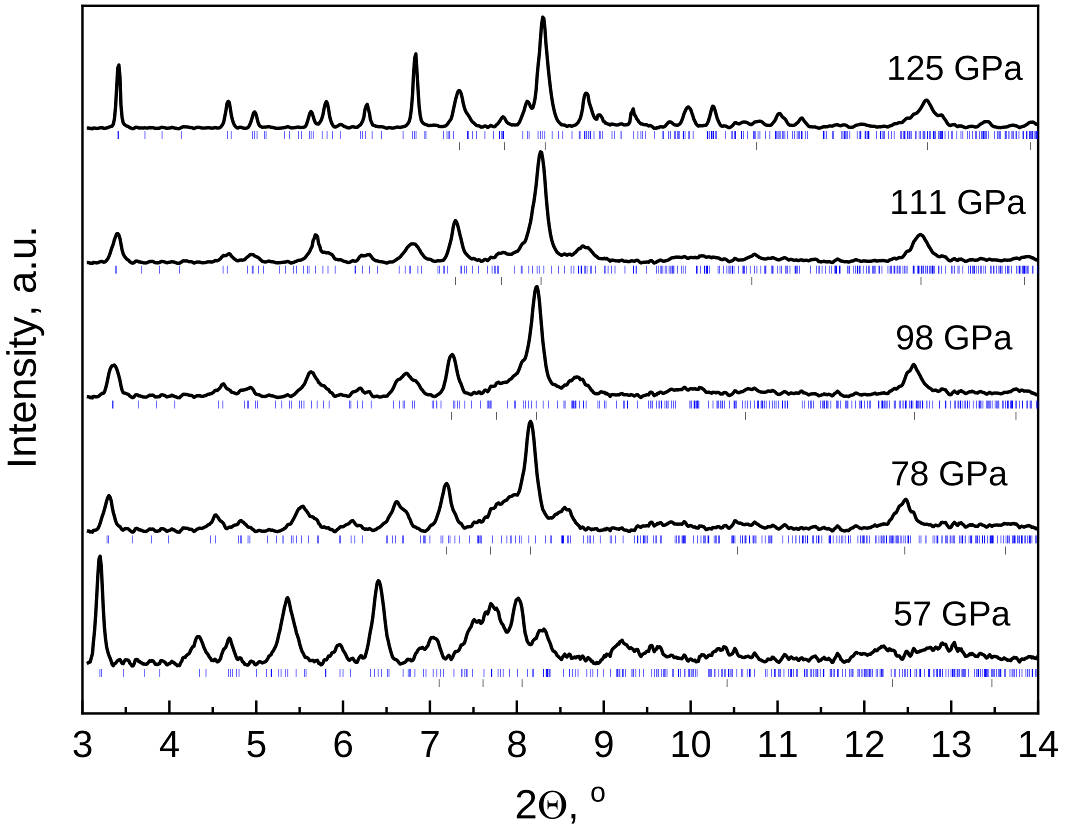

Fig. S2. Background-subtracted X-ray diffraction profiles (λ = 0.2846 Å) from the sample collected on decompression. The blue tick marks represent the position of the $Y(N_5)_3{\cdot}N_2$ reflections; the black tick marks correspond to the reflections of Re from the gasket. After the first decompression step, the crystallite's quality significantly decreases, leading to peak broadening and a significant decrease in the intensity of the single crystal reflections at high 2θ angles. Nevertheless, the strong reflections of

$Y(N_5)_3{\cdot}N_2$ in 2θ = 3-9° range were observed down to the last collected pressure point of 57(2) GPa. The intensity ratios of diffraction lines in the pattern collected at 57(2) GPa differ from those at higher pressures, due to differently oriented $Y(N_5)_3{\cdot}N_2$ grains; this pattern was recorded at a different location in the sample chamber, where the best-quality crystallites were present at 57(2) GPa. No secondary phases were detected at any pressure step.

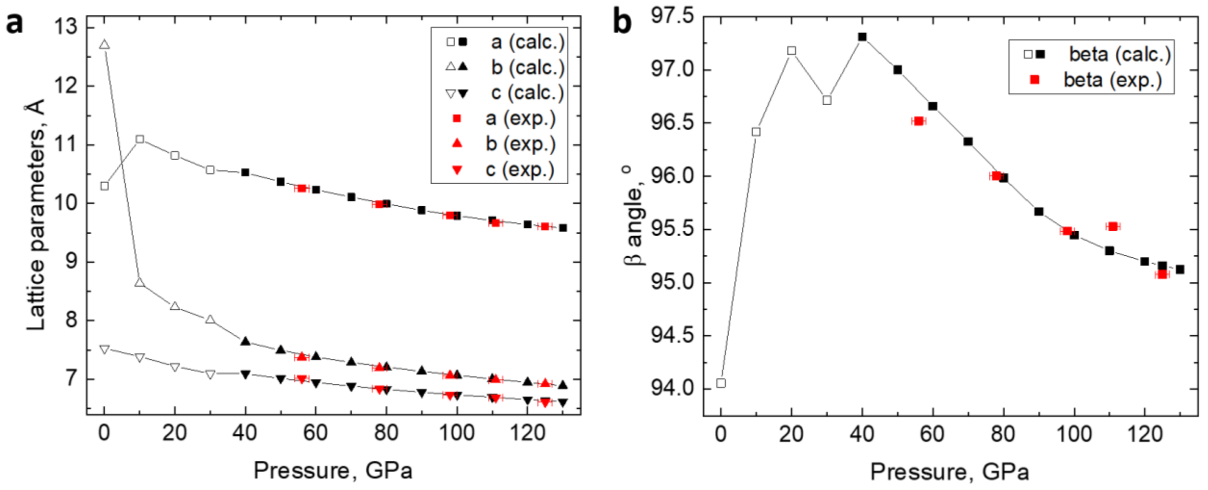

Fig. S3. The dependence of the unit cell parameters on the pressure of $Y(N_5)_3{\cdot}N_2$: (a) *a, b, c* lattice parameters and (b) *β* angle. The black outline and filled symbols represent calculated data points obtained from DFT (PBE GGA), the red symbols represent experimental data points obtained from XRD data. According to PBE DFT calculations, the lattice parameters of $Y(N_5)_3{\cdot}N_2$ change monotonously down to 40 GPa, then between 40 and 30 GPa a notable hop of *a*, *b*, *c*, and *β* is notable. In the 30-10 GPa range, they again change smoothly and the relaxation at ambient pressure resulted in enormous changes of *a*, *b*, and *β*.

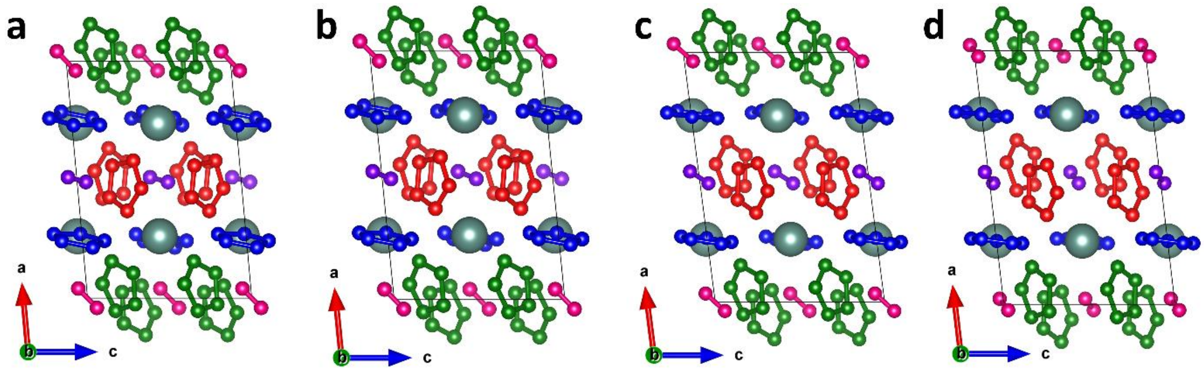

Fig. S4. The DFT-relaxed (PBE GGA) structures of $Y(N_5)_3{\cdot}N_2$ viewed along *b*-direction at (a) 125 GPa, (b) 90 GPa, (c) 40 GPa, (d) 30 GPa. The crystal structure does not undergo any significant changes down to 40 GPa; between 40 and 30 GPa one can see the significant rotation of both dimers and dimers keep this orientation down to 10 GPa. At 1 atm the relaxed structure is completely different from the initial one.

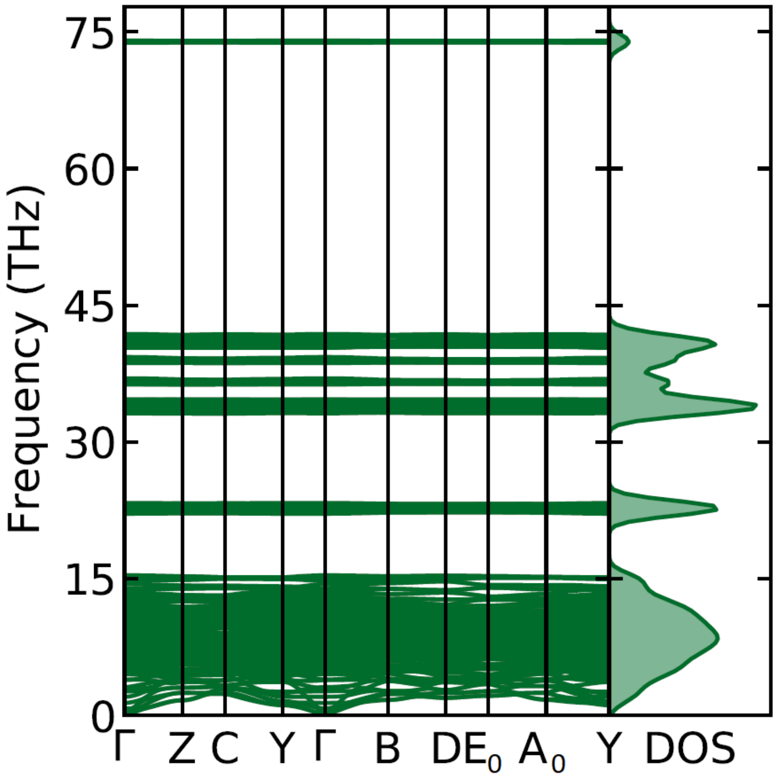

Fig. S5. Phonon dispersion curves and phonon density of states for $Y(N_5)_3{\cdot}N_2$ at 40 GPa. The absence of imaginary modes demonstrates the dynamical stability $Y(N_5)_3{\cdot}N_2$ at 40 GPa.

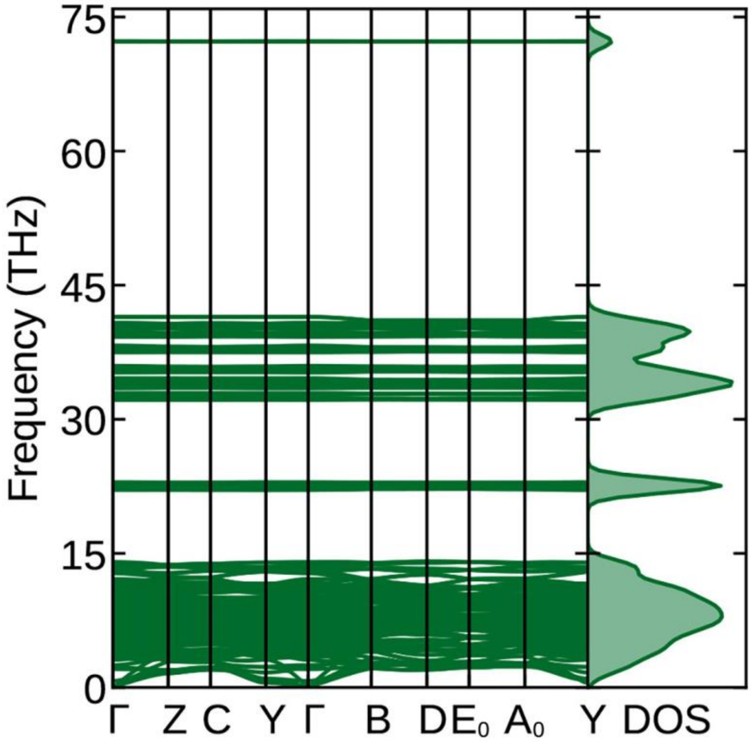

Fig. S6. Phonon dispersion curves and phonon density of states for the DFT-relaxed $Y(N_5)_3{\cdot}N_2$ model with rotated N_2 dimers at 30 GPa. The absence of imaginary modes demonstrates its dynamical stability at 30 GPa.

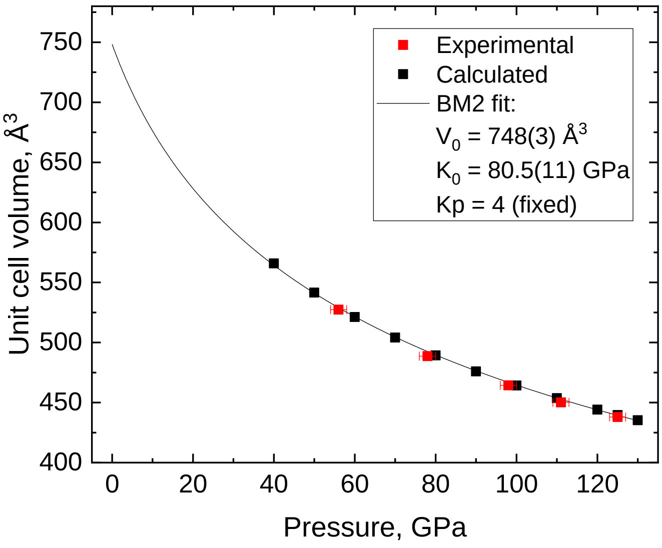

Fig. S7. Experimental (red points) and calculated (black points) pressure dependence of the $Y(N_5)_3{\cdot}N_2$ unit cell volume. The black curve is the fit of the calculated P-V data using a 2nd order Birch-Murnaghan equation of state, yielding K_0=80.5(11) GPa, K'= 4 (fixed), and V_0=748(3) $Å^3$. It also can be fitted using a 3rd order Birch-Murnaghan equation of state, yielding K_0=55.5(9) GPa, K'= 4.46(2), and V_0=793(2) $Å^3$.

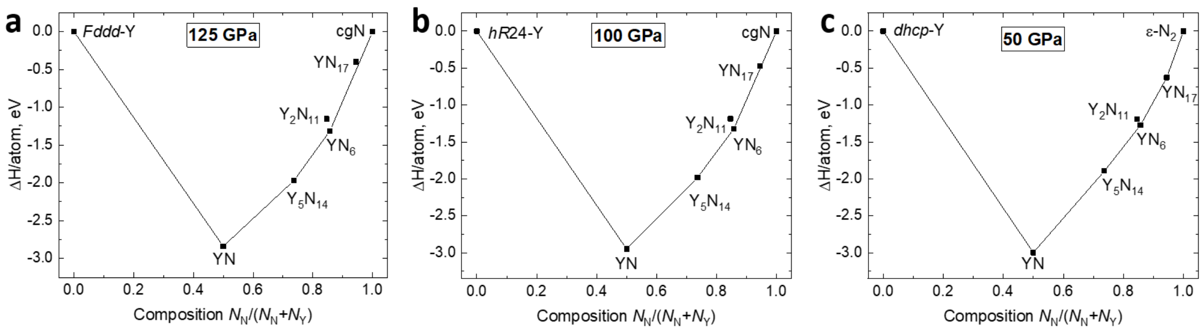

Fig. S8. The calculated convex hulls in the Y-N binary system at (a) 125 GPa, (b) 100 GPa, and (c) 50 GPa. Only experimentally confirmed binary Y-N phases were used in the calculations. Static enthalpies were calculated for Y (*Fddd*, *hR*24 or *dhcp* allotropes[23]), nitrogen (*cg*-N or ε-N_2 allotropes[24,25]), YN (rocksalt structure), Y_5N_{14},[26] YN_6,[27] Y_2N_{11},[27] and $Y(N_5)_3{\cdot}N_2$ (denoted as YN_{17}) from the current study. At 125 GPa YN_{17} is 113 meV/atom above the convex hull. At 100 GPa YN_{17} is 43 meV/atom above the convex hull. At 50 GPa YN_{17} lays on the convex hull.

References

[1] I. Kantor, V. Prakapenka, A. Kantor, P. Dera, A. Kurnosov, S. Sinogeikin, N. Dubrovinskaia, L. Dubrovinsky, *Rev. Sci. Instrum.* **2012**, *83*, 125102.

[2] R. Boehler, *Rev. Sci. Instrum.* **2006**, *77*, 2004–2007.

[3] A. Kurnosov, I. Kantor, T. Boffa-Ballaran, S. Lindhardt, L. Dubrovinsky, A. Kuznetsov, B. H. Zehnder, *Rev. Sci. Instrum.* **2008**, *79*, 045110.

[4] T. Fedotenko, L. Dubrovinsky, G. Aprilis, E. Koemets, A. Snigirev, I. Snigireva, A. Barannikov, P. Ershov, F. Cova, M. Hanfland, N. Dubrovinskaia, *Rev. Sci. Instrum.* **2019**, *90*, 104501.

[5] Y. Akahama, H. Kawamura, *J. Appl. Phys.* **2006**, *100*, 043516.

[6] S. Anzellini, A. Dewaele, F. Occelli, P. Loubeyre, M. Mezouar, *J. Appl. Phys.* **2014**, *115*, 043511.

[7] *Rigaku Oxford Diffraction, CrysAlisPro Softw. Syst.* **2015**.

[8] A. Aslandukov, M. Aslandukov, N. Dubrovinskaia, L. Dubrovinsky, *J. Appl. Crystallogr.* **2022**, *55*, 1383–1391.

[9] O. V. Dolomanov, L. J. Bourhis, R. J. Gildea, J. A. K. Howard, H. Puschmann, *J. Appl. Crystallogr.* **2009**, *42*, 339–341.

[10] V. Petrícek, M. Dušek, L. Palatinus, *Zeitschrift fur Krist.* **2014**, *229*, 345–352.

[11] G. M. Sheldrick, *Acta Crystallogr. Sect. C Struct. Chem.* **2015**, *71*, 3–8.

[12] K. Momma, F. Izumi, *J. Appl. Crystallogr.* **2011**, *44*, 1272–1276.

[13] G. Kresse, J. Furthmüller, *Phys. Rev. B* **1996**, *54*, 11169–11186.

[14] G. Kresse, D. Joubert, *Phys. Rev. B - Condens. Matter Mater. Phys.* **1999**, *59*, 1758–1775.

[15] J. P. Perdew, K. Burke, M. Ernzerhof, *Phys. Rev. Lett.* **1996**, *77*, 3865–3868.

[16] M. Zhou, M. Sui, X. Shi, Z. Zhao, L. Guo, B. Liu, R. Liu, P. Wang, B. Liu, *J. Phys. Chem. C* **2020**, *124*, 11825–11830.

[17] M. Zhou, S. Liu, M. Du, X. Shi, Z. Zhao, L. Guo, B. Liu, R. Liu, P. Wang, B. Liu, *J. Phys. Chem. C* **2020**, *124*, 19904–19910.

[18] M. Bykov, E. Bykova, S. Chariton, V. B. Prakapenka, I. G. Batyrev, M. F. Mahmood, A. F. Goncharov, *Dalt. Trans.* **2021**, *50*, 7229–7237.

[19] B. A. Steele, E. Stavrou, J. C. Crowhurst, J. M. Zaug, V. B. Prakapenka, I. I. Oleynik, *Chem. Mater.* **2017**, *29*, 735–741.

[20] Y. Xu, L. Tian, D. Li, P. Wang, M. Lu, *J. Mater. Chem. A* **2019**, *7*, 12468–12479.

[21] Y. Xu, L. Ding, F. Yang, D. Li, P. Wang, Q. Lin, M. Lu, *Chem. Eng. J.* **2022**, *429*, 132399.

[22] Y. Xu, Q. Lin, P. Wang, M. Lu, *Chem. - An Asian J.* **2018**, *13*, 1669–1673.

[23] E. J. Pace, S. E. Finnegan, C. V. Storm, M. Stevenson, M. I. McMahon, S. G. MacLeod, E. Plekhanov, N. Bonini, C. Weber, *Phys. Rev. B* **2020**, *102*, 094104.

[24] M. I. Eremets, A. G. Gavriliuk, I. A. Trojan, D. A. Dzivenko, R. Boehler, *Nat. Mater.* **2004**, *3*, 558–563.

[25] R. L. Mills, B. Olinger, D. T. Cromer, *J. Chem. Phys.* **1986**, *84*, 2837–2845.

[26] A. Aslandukov, A. Aslandukova, D. Laniel, I. Koemets, T. Fedotenko, L. Yuan, G. Steinle-Neumann, K. Glazyrin, M. Hanfland, L. Dubrovinsky, N. Dubrovinskaia, *J. Phys. Chem. C* **2021**, *125*, 18077−18084.

[27] A. Aslandukov, F. Trybel, A. Aslandukova, D. Laniel, T. Fedotenko, S. Khandarkhaeva, G. Aprilis, C. Giacobbe, E. Lawrence Bright, I. A. Abrikosov, L. Dubrovinsky, N. Dubrovinskaia, *Angew. Chemie Int. Ed.* **2022**, *61*, e202207469.
